# Domestication and Function

**Published:** 1888-07

**Authors:** Cornelius M. O’Leary

**Affiliations:** Manhattan College, New York


### Art. XVIII.—DOMESTICATION AND FUNCTION.

BY CORNELIUS M. O'LEARY, M.D., LL.D.
*Manhattan College, New York.*

No known influence is so powerful in effecting radical changes in the habits of animals as domestication. The results which depend upon the natural selection are so gradual that they can be worked out only with the lapse of centuries, and, consequently, can never fall under observation, whereas the consequences of domestication are rapid and striking, and the contrasts between the same species in the feral state and as molded by the mastery of man, are at all times an easy matter of study and observation. The assignment of these changes to their proper cause, the discovery of the conditions on which depend profound differences in functional activity without any apparent structural change, suggest a line of inquiry that cannot fail to lead to interesting and instructive conclusions. If we compare the Quagga of South Africa with the plodding donkey of civilized countries, we will not fail to be impressed with the close anatomical resemblances that exist between them, even to the absence of the small callosity on the inner side of the hind leg, which is a distinctive mark of the horse. And yet what remarkable differences in habits and disposition between both congeners ! The one fierce, fleet and untamable, a true child of the desert, the other patient, meek and docile, even to a proverbial extent. It is plain that to the effects of domestication alone we must attribute those sharp differences of habit and disposition which mark off the *asinus vulgaris* from the wild ass of Abyssinia, from which the former is supposed by Darwin to be descended. It is not on the organs, however, that these differences have been imposed, for, however much these may have been subjected to variety of function, they still remain substantially identical as they exist in the wretched specimens to be found in the north of India, in the light and graceful asses of the Arabs and the Persians, and in the wild herds of Mesopotamia. If the principle of natural selection as known to us were in exclusive operation in the case of these animals, we would see marked structural differences ensuing upon the continued diversity of function, since, according to the fundamental notion of evolution, it is in diversity of function that anatomical changes take their rise. Domestication, therefore, may be viewed as a remote and distinct step in the progress of selection, different from those which in other cases have led to a branching off from primitive types, yet essentially similar, since it is destined eventually to lead to the same results.

The facts of domestication, therefore, must prove of interest to naturalists as being calculated to throw light upon what may be regarded as a tentative departure from prevailing types, ere yet any structural peculiarities have been developed. Domestication is of a two-fold character as respects the results which it furnishes, for it shows us that, under the influence of man's training, animals lose certain traits or instincts and acquire new ones; in a word, it is both positive and negative. The loss of ferocity in the cat is simply marvelous when we reflect upon the savage tempers of the tiger and the catamount, which were unquestionably the ancestral prototypes of the purring tabby. In certain parts of Germany, according to Stuorn, as quoted by Romanes, where for many generations it has been the custom to remove calves from their mothers immediately after birth, the maternal instincts have almost entirely disappeared in the cattle. The positive aspects of domestication are still more numerous and impressive. One of these is curiously illustrated in the sense of ownership which has been developed in the dog. Not only will this sagacious animal lose its life in defence of its owner's property, but it delights to manifest the fact that it is itself the chattel property of man. It even recognizes the validity of a transfer of ownership, and assumes allegiance to its master's friend to whose care it may have been assigned, with alacrity and fidelity. Pointers, setters and retrievers also exhibit, each in its peculiar line of acquired capacity, a striking illustration of some of the positive results of domestication. The first thing which impresses us in studying the effects of domestication on animals is that, though the environments and mode of life may have been profoundly modified, such modification has so far failed to develop any essential change in the anatomical structure of any individual so as to remove it from the same species to which individuals in the wild state belong. Horses, pigs, goats, sheep, in a word, all those animals which man has made obedient to his will, and on which he has imposed every possible modification which ever-changing environments suggested, still retain the essential anatomical characters of their untamed primitive types. However much function may have induced structural changes through the operation of natural selection, at least domestication has exhibited no such result. Still this condition cannot be said to be without some influence on some portion of the system, since we know that the habits and disposition of domesticated animals are transmitted and even intensified by heredity, and that consequently the causes of these must be organically established in the constitution. Since, however, on the one hand, no structural change has been wrought through the operation of domestication, and, on the other, we know that habits and dispositions, inasmuch as they are physical facts, must be referred to the nervous system, it follows that in the nervous centres we must look for an explanation of the phenomena which domestication exhibits. Every emotion becomes, as it were, incarnated in the nerve centre which has been thrown into excitation on the occasion of the emotional activity, and leaves behind it permanent vestiges of its energy. This organic modification of the nerve cell is purely molecular, similar to the change which occurs to the molecules of luminous ether in the process of polarization. Of course, no microscope even can reveal to us the existence of such a change, since it is a mere change in the relative position of the atoms of the molecule. Still this change, subtle as it is, is transmitted just as are the specific anatomic characters of the parent, and produce in the offspring the same habits and disposition with which they were first associated. But mere molecular changes do not continue forever without generating other changes that extend to the nerve tissues and reveal themselves under the microscope. These changes, however, are not as yet appreciable, and we find in this fact an evidence that those processes of nature that are destined to lead to organic changes and aberration from prevailing types are essentially slow, and cannot be effected short of epochs that cover almost countless thousands of years. We know that domestic animals have been under man's dominion from time immemorial, and yet we fail to discover any departure from a feral type of ancestry except such as is accidental and retrievable. How many eons, therefore, must it not have required the principle of natural selection to have been in operation ere it elaborated those profound departures from primitive types which mark the specific and generic changes of living beings, since thousands of years have been insufficient to determine even an approach to such changes through the intervention of artificial selection.

First approaches to a departure from a given type originate in the nervous centres and culminate in permanent structural changes in the anatomical tissues generally. Thus the new habits and dispositions which domestication has engendered in the dog or in the horse are, if the theory of evolution has any claim to a substantial basis, the forerunners to those modifications which are to result in the assumption by either of the characters and capacities that will mark their assimilation to a different species. They have their seat in the different nervous centres which control the functions with which they are connected, and imply either a cessation of functional activity in the nerve cells, when lapsing instincts are the result, or a changed functional activity where the instincts have undergone a profound alteration This implies the cessation of previous currents that existed between the optic thalamus and the psycho-motor zones of the cortical periphery of the brain, or the establishment of new currents between these same points. These currents may be said to mark the first effects of domestication or to be the initial steps in the acquisition of new habits and dispositions, and to be consequently the first link in the chain of physiological and anatomical changes which result in the final operation of natural selection. Thus it will be seen that so far as reasoning on hypotheses possesses any validity, domestication may be viewed as the co-efficient of the agencies that wrought the first results that have led to permanent changes of type, only that, so far, it has produced no appreciable structural or organic alteration. If this, then, be so, we may infer that under purely natural conditions, that is, where the influence of domestication has not come into play, a change of habits and disposition were the first consequences of changed environment. A change in the nerve currents between the ganglia contained in the optic thalamus and the cortical periphery or grey matter of the brain, may be presumed to follow such a change, new currents being established and previously existing ones ceasing to act. After this the molecular changes in the nerve cells are supplemented by structural changes appreciable by the aid of the microscope, and portend such structural changes as are to be followed by permanently altered function. The first structural change, however, must originate in the nerve cells, since it was their altered sympathies that engendered the habits and dispositions acquired through the agency of domestication. Moreover, it is a principle in physiology that the character of an organic growth is determined by the quality of the innervation which supplies a given part of the system. Now, if an acquired habit entails a change in the use of some bodily organ, a previous influence must have been experienced by the special ganglion which presided over that organ in the course of its prior activities. Consequent upon this change in the character of a nerve cell, or rather, a group of nerve cells, we will have incipient traces of structural change in the organ over which that group presided. Thus, if the cells which control the emotional tendencies that develop ferocity are kept in a state of functional abeyance through fear, the animal, which is made the subject of the experiment, gradually loses its savage character, and acquires a more docile disposition, and at the same time, the polarity, so to speak, of the cells, becomes altered, a molecular change is wrought in them and they adapt themselves to new incoming and outgoing discharges of nervous force. This change may be called sympathetic, since it is the result of a law in accordance with which all organisms adapt themselves to an atmosphere of new conditions. But this purely sympathetic change must at some time merge into an organic fixity, and then a change of structure begins to manifest itself in the nerve tissue, and we here find the first anatomical transformation that altered environments produce. Once granted, this structural modification in the nervous texture, we will find no difficulty in accounting for the more obvious and striking structural changes to be found in the bodily organs. If the nervous innutrition which supplied the unwebbed foot of a hen be changed owing to some structural change in the ganglion whence its innervation was derived, we can easily conceive an organic change to a web foot. To sum up, then, the views somewhat crudely presented in this paper, and which claim no more than such conjectural validity as belongs to a mere hypothesis, domestication furnishes a partial basis for the surmise that the law of evolution works out its result in the animal kingdom somewhat after this fashion. 1. Changed habits and disposition. 2. Change of function. 3. Structural change in nerve cells 4. Permanent change in bodily organs. 5. Transition of type.

